# Malaria patient spectrum representation in therapeutic clinical trials of uncomplicated malaria: a scoping review of the literature

**DOI:** 10.1186/s12936-023-04441-5

**Published:** 2023-02-10

**Authors:** Lorenzo Arena, Mazvita Zanamwe, Christine M. Halleux, Verena Carrara, Brian J. Angus, Proochista Ariana, Georgina S. Humphreys, Caitlin Richmond, Kasia Stepniewska, Philippe J. Guérin, Piero L. Olliaro

**Affiliations:** 1 Centre for Tropical Medicine and Global Health, Nuffield Department of Medicine, University of Oxford, Oxford, UK; 2 WorldWide Antimalarial Resistance Network (WWARN), Oxford, UK; 3 Infectious Diseases Data Observatory (IDDO), Oxford, UK; 4 Special Programme for Research and Training in Tropical Diseases (TDR), World Health Organization (WHO), Geneva, Switzerland; 5 Institute of Global Health, Faculty of Medicine, University of Geneva, Geneva, Switzerland; 6 ISARIC Global Support Centre, International Severe Acute Respiratory and Emerging Infection Consortium, Pandemic Sciences Institute, University of Oxford, Oxford, UK

**Keywords:** Malaria, Antimalarials, Clinical trial, Patient selection, Review, Uncomplicated malaria

## Abstract

**Background:**

For the results of clinical trials to have external validity, the patients included in the study must be representative of the population presenting in the general clinical settings. A scoping literature review was performed to evaluate how the eligibility criteria used in anti-malarial efficacy and safety trials translate into patient selection.

**Methods:**

A search of the WorldWide Antimalarial Resistance Network (WWARN) Clinical Trials Publication Library, MEDLINE, The Cochrane Library, and clinicaltrials.gov was conducted to identify trials investigating anti-malarial efficacy and safety, published between 14th April 2001 and 31st December 2017. An updated search using the WWARN Clinical Trial Publication Library was undertaken to identify eligible publications from 1st January 2018 to 31st July 2021. The review included studies in patients of any age with uncomplicated malaria and any pharmaceutical therapeutic intervention administered. The proportion of trials with malaria-positive patients excluded was calculated and linked to the reported reason for exclusion. A subgroup analysis on eligibility criteria and trial baseline demographics was conducted to assess whether criteria are complied with when recruiting patients.

**Results:**

Out of 847 studies, 176 (21%) trials were included in the final synthesis, screening a total of 157,516 malaria-positive patients, of whom 56,293 (36%) were enrolled and treated. Across the 176 studies included, 84 different inclusion and exclusion criteria were identified. The reason for exclusion of patients who tested positive for malaria was reported in 144 (82%) studies. Three criteria account for about 70% of malaria-positive patients excluded: mixed-species malaria infections or other specific *Plasmodium* species, parasite counts outside the set study ranges, and refusal of consent.

**Conclusions:**

Nearly two-thirds of the malaria-positive subjects who present to health facilities are systematically excluded from anti-malarial treatment trials. Reasons for exclusions are largely under-reported. Anti-malarial treatment in the general population is informed by studies on a narrow selection of patients who do not fully represent the totality of those seeking antimalarial treatment in routine practice. While entry criteria ensure consistency across trials, pragmatic trials are also necessary to supplement the information currently available and improve the external validity of the findings of malaria clinical trials.

**Supplementary Information:**

The online version contains supplementary material available at 10.1186/s12936-023-04441-5.

## Background

Despite significant successes in malaria control, over 200 million malaria cases still occur globally every year, requiring treatment [1]. Guidelines for the management of clinical malaria are largely based on empirical evidence from clinical efficacy trials [2].

Standardizing trial methodologies is important for comparability across sites and over time [3]_,_ especially when monitoring efficacy for possible parasite resistance, and facilitates aggregating and meta-analysing findings [3–5]. Eligibility criteria tend to be stricter during the clinical development of a new medicine (phase II-III clinical trials) but are expected to become more inclusive as knowledge of the treatment increases, in post-registration (phase IV) studies. However, if clinical trials are systematically selective in their eligibility criteria, this may result in a proportional under-representation of certain segments of the patient population seen in clinical practice and limit the generalizability of the findings in the general population [5]. When determining the programmatic effectiveness of a treatment, trial methodology, notably eligibility criteria, should be adapted to reflect the full range of the malaria patients seen in clinical practice.

The World Health Organization (WHO) guidelines for the assessment of anti-malarial drug efficacy in falciparum malaria (see Box 1) are geared towards ensuring that treatments are consistently tested to measure efficacy and monitor parasite resistance [6]. A different question is whether these trials also provide a comprehensive picture of the efficacy and safety of treatments across the range of malaria patients routinely seen in the clinics.

**Box 1:** WHO eligibility criteria [3]

Age under 5 years (as they are the most vulnerable population, and the ones where immunity is least likely to play a role in parasite and fever clearance) [7];
parasitologically-confirmed infection, parasite range of 1000–100,000 asexual parasites/µl for low to moderate transmission, and 2000 to 200,000 asexual parasites/µl for areas of high transmission (depending on local transmission intensities); measured axillary temperature ≥ 37.5 °C or history of fever within the last 24 h; and the exclusion of patients with danger signs of severe malaria, severe malnutrition, pregnancy and lactation, and chronic severe illness

This scoping review was conducted to assess the representativeness of the study participants enrolled in malaria clinical trials vis-à-vis the general population by identifying the reasons for excluding patients who had tested malaria-positive from the published literature and calculating the proportion of patients excluded.

## Methods

The Preferred Reporting Items for Systematic reviews and Meta-Analyses extension (PRISMA) methodology was used [8].

### Literature search

Data for this review were identified through searches of MEDLINE, the WorldWide Antimalarial Research Network (WWARN) Clinical Trials Publication Library [9], The Cochrane Library, clinicaltrials.gov, and manually examined bibliographic references from relevant articles using the search terms “malaria” AND “therapy” OR “treatment” OR “therapy” OR “therapeutics”. Literature search strategies were developed using the medical subject headings (MeSH) terms and text words related to uncomplicated malaria pharmaceutical therapy. Search terms and conditions are provided as supplementary information (see Additional file 3). The initial search was limited to trials published between 14th April 2001 and 31st December 2017. The starting date was selected as it correlated with the publication of the first Consolidated Standards of Reporting Trials (CONSORT) statement [10]. Articles were limited to those published in English, Portuguese, Spanish, or French and reviewed for their adherence to the CONSORT statement and reporting of malaria clinical trial eligibility criteria.

An update search using the WWARN Clinical Trial Publication Library, a living systematic review of all malaria clinical efficacy trials [11], was conducted to identify eligible studies published from 1st January 2018 to the latest update available, 31st July 2021.

### Study selection

Studies identified from the search were screened for eligibility using the following criteria. A study was included if it met all the criteria.

- a non-comparative or comparative design (randomized controlled trial (RCT), cluster RCT, or controlled (non-randomized) clinical trial);
- uncomplicated symptomatic malaria, caused by parasitologically-confirmed *Plasmodium falciparum, Plasmodium vivax, Plasmodium malariae, Plasmodium ovale,* or *Plasmodium knowlesi*;
- a CONSORT flow diagram [12], or similar adaptation, available with the *total number of patients screened,* the *total number of malaria-positive patients* and the *total number of malaria-positive patients excluded*;

There were no restrictions on study settings or age of the patients enrolled. Studies were excluded if they met one of the following criteria: phase 1 (healthy volunteers), studies on severe malaria, cross-sectional studies, case-series and case-reports, meta-analyses, observational studies, studies on prevention, prophylaxis, or animal studies.

### Data extraction and management

Data extraction from the initial search was conducted by one researcher (MZ), using a detailed predefined variable dictionary and partially checked by two other researchers (CH, PLO). Extraction of data from eligible studies identified in the update search was completed by one researcher (LA) and validated by another researcher (VC) using the same predefined variable dictionary. To ensure consistency across the totality of extracted data, two researchers (LA, VC) reviewed and validated the information extracted from the initial search. Information on study inclusion and exclusion criteria, number of patients excluded for each criterion were extracted from the text and the CONSORT flow diagram, and data on population demographics were extracted from tables and text. Information was recorded on a standardized REDCap database [13, 14] and analysed with R studio and Excel. Source data used for the analysis, and the associated dictionary are available as Additional files 1 and 2.

Further information on the recording of the data items, baseline demographics, and the subsequent subgroup narrative analysis can be found in Additional files 2 and 7.

## Results

### Search results, number of eligible articles

**Fig. 1:**
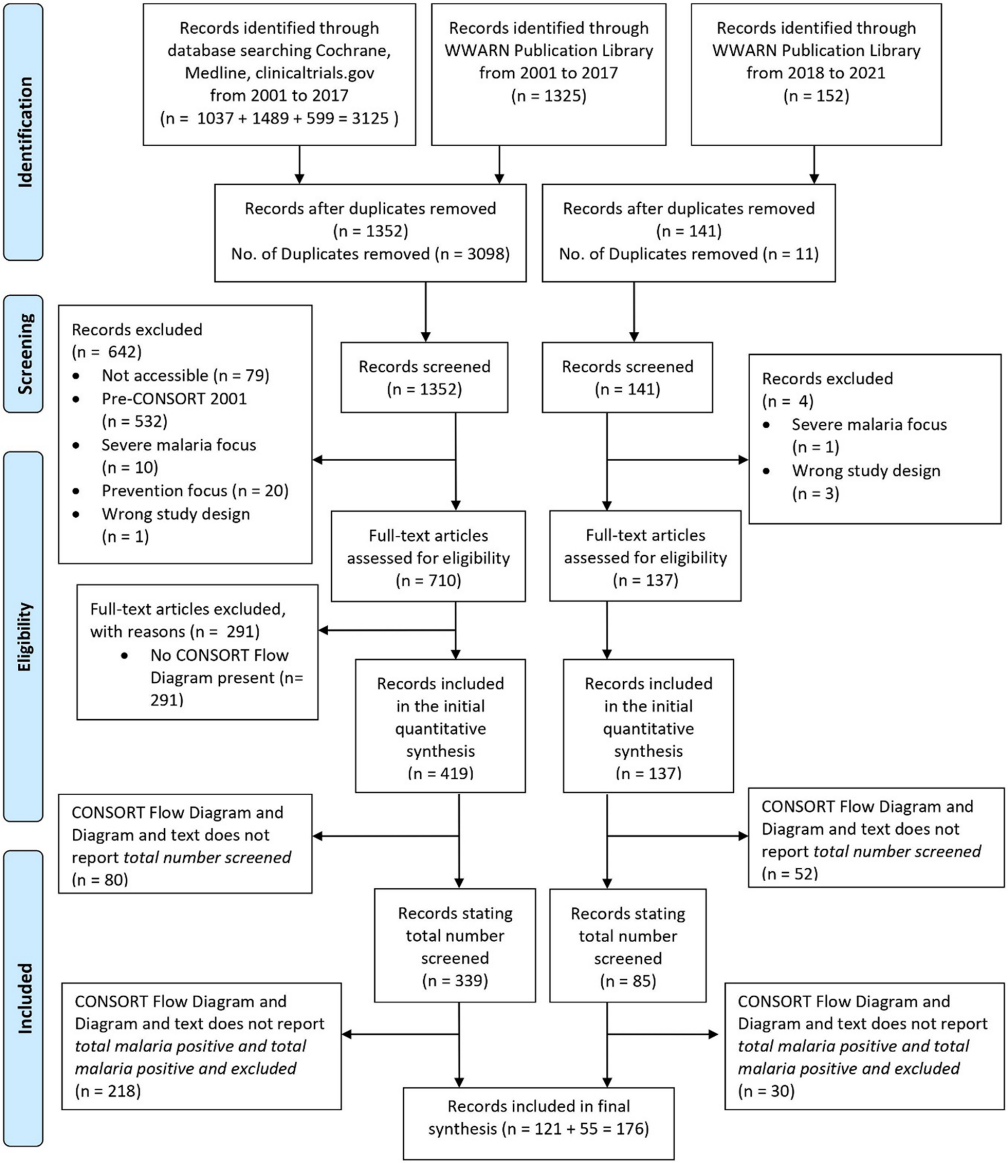
PRISMA flow chart representing the number of items identified in the search and subsequently screened and assessed for eligibility. The search strategy and eligibility criteria are summarized in the Methods section and further details are given in the supplementary information

Of the 1493 articles screened by title and abstract (Fig. 1), 646 were excluded. Of these excluded, 532 were published prior to the CONSORT 2001 cut-off date (14th April 2001); 79 full texts were not accessible, 11 focused on severe malaria, 20 focused on prevention, and 4 contained ineligible study designs. The remaining 847 full-text articles were accessed and assessed for eligibility. Of these, 291 (34%) were excluded for not providing sufficient information on eligibility and exclusions (such as absence of the CONSORT Flow Diagram or similar adaptation in the manuscript), and/or not meeting other inclusion criteria. From the remaining 556 records, a further 132 publications were excluded from the analysis as they did not provide *the total number of subjects screened*, and another 248 for not reporting the *total number of malaria-positive* screened as well as the *total number of malaria-positive and excluded* screened patients from the clinical trial. A total of 176 records were retained in the final synthesis (see Additional file 9), accounting for 157,516 malaria-positive patients diagnosed of whom 56,293 were enrolled and treated. These 176 papers represent 21% of the 847 identified on the treatment of uncomplicated malaria published after the CONSORT 2001 statement. The species and geographical distribution of studies are summarized in Table 2.

**Table 1:** Number of articles reviewed by species and WHO region inclusion criteria

WHO Region							
	African	South-East Asia	Americas	Western Pacific	Eastern Mediterranean	Global^a^	Total per species (%)
*P. falciparum*	98	31	3	1	0	1	134 (76)
*P. vivax*	7	9	2	0	3	2	23 (13)
*P. knowlesi*	0	1	0	0	0	0	1 (1)
Mixed species	5	11	0	1	0	1	18 (10)
Total regional distribution (%)	110 (62.5)	52 (30)	5 (3)	2 (1)	3 (2)	4 (2)	176

^a^When a trial is conducted in multiple sites from different WHO regions

The median number of studies per year was 8.5

Additional file 8 reports the number of studies published by year between January 2001 and July 2021

### Study eligibility criteria

Across the 176 eligible publications, 48 (27%) referenced either WHO guidelines [3, 15–17] for the assessment of anti-malarial drug efficacy in falciparum malaria (90%) or other protocols [18–20] for the definition of the eligibility criteria (see Box 1). The definitions of the study inclusion and exclusion criteria extracted from each study are described in Additional file 2.

**Fig. 2:**
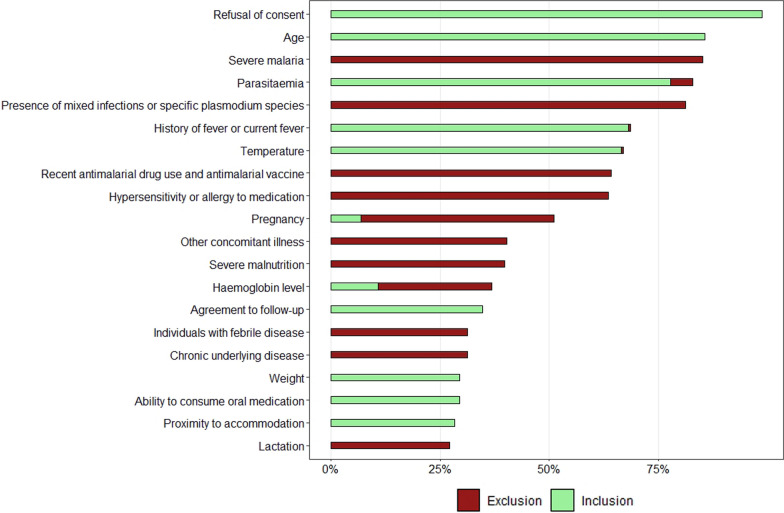
Frequencies of inclusion and exclusion criteria reported in at least 25% of the 176 studies included in the final analysis. Criteria could be reported as inclusion (green) as well as exclusion (red), according to the publication. For example, accepted parasitaemia ranges could be reported either as range within which patients are eligible for inclusion or as cut-off values below or above which patients are excluded

Eighty-four (84) eligibility criteria were identified, 17 inclusions and 77 exclusions, with ten criteria reported in either inclusion or exclusion eligibility sections, five of which (parasitaemia, history of fever or current fever, temperature, pregnancy, haemoglobin level) are described in Fig. 2 and the remaining in Additional file 4. Twenty eligibility criteria were commonly reported across 25% of the publications with a large proportion of the trials requiring consent (n = 174, 99%) of the patient or guardian to be enrolled in the trial. Two of the eligible trials did not explicitly report having requested consent to enrol patients in the study. As inclusion criteria, out of the 176 studies, 151 of the eligible studies (86%) required specific age ranges for inclusion, 121 (69%) had history of fever or current fever, or in 118 (67%) documentation of body temperature above a predefined cut-off. Ability to comply with the study follow-up, ability to consume oral medication and specific weight ranges, and proximity to the study centre, were reported in 61 (35%), 52 (30%), 52 (30%), respectively. As exclusion criteria, 150 studies (85%) excluded patients with signs and symptoms of severe malaria (123, 82%), 15 (10%), 11 (7%) and 1 (0.7%) studies for *P. falciparum*, mixed, *P. vivax* and *P. knowlesi* trials, respectively. Patients with mixed infections or other specific *Plasmodium* species (143/176, 81%), recent use of an anti-malarial (113, 64%), allergy to medication (112, 64%) or pregnancy (78, 44%) were commonly excluded. Accepted parasitaemia ranges were reported as either inclusion in 137 (78%) studies or as exclusion criteria (i.e. values outside which a patient is not eligible) in 9 (5%) studies (Fig. 2).

#### Parasitaemia

All 176 studies specified the diagnostic method to confirm malaria; microscopy alone was the most common (104, 59% of studies); combinations of microscopy and Polymerase Chain Reaction (PCR) or Rapid Diagnostic Test (RDT) were used in 37 (21%) and 29 (16%) studies, respectively. A combination of microscopy, PCR and RDT were reported in 4 (2%) studies. RDT or PCR alone was reported in 1 study each (0.6%).

Parasitaemia inclusion ranges were described in 110/134 (82%), 15/23 (65%), 1/1 (100%) of the pure *P. falciparum*, *P. vivax* and *P. knowlesi* studies, respectively. Of the 110 *P. falciparum* studies, 50 (45%) had an upper limit of 200,000 asexual parasites/µl, and 22 (20%) had a range of 2000–200,000. Of the 15 *P. vivax* studies, 14 (93%) required having  ≥ 250 asexual parasites/µl while 10/14 (71%) of mixed-infection studies included different parasitaemia ranges for the different *Plasmodium* species. Less frequently used criteria involve the presence of gametocytes (8/176 studies, 5.0%), and second malaria episode or symptomatic malaria (1/176, 0.6%).

#### Demographics

(i) One hundred thirty-three (76%) of the eligible studies were conducted in children ≥ 6 months old and above and/or adults; (ii) 17/% (30/176) enrolled only children between 0 and 5 years old; (iii) 21/176 (12%) enrolled only 6–59 month-old; (iv) 63/176 (36%) enrolled subjects ≥ 6 months with no upper age limit. A broad range of other ages was used with various frequencies in the reminding studies (Additional file 9).

Forty-nine (28%) trials included participants who weighed more than 5 kg, 46 of which with no upper weight limit. Two (1%) studies restricted the inclusion only to male participants.

#### Fever

Documentation of body temperature above a predefined cut-off temperature, typically 37.5 °C or 38 °C (Additional file 10), was reported in 113/176 (64%) of studies; 97/176 (55%) required both history of fever or current fever temperature above a predefined cut-off; 23/176 required history of fever or current fever alone.

#### Pregnancy

Twelve (7%) studies included pregnant women with a minimum gestational age of 12 weeks. None included women in the first trimester of pregnancy. Other reasons for exclusion associated with pregnancy were: lactation (48/176,) multiple pregnancies (4/176), women of reproductive age (3/176), previous history of complicated pregnancy (2/176) or abortion (2/176), planning pregnancy (1/176), missed menstrual cycle (1/176) or currently menstruating (1/176).

#### Concomitant illness

Patients with unspecified concomitant illness, severe malnutrition, or chronic underlying diseases were excluded in 71 (40%), 70 (40%), and 55 (31%) publications, respectively. 50/176 of the trials that excluded subjects with severe malnutrition were located in the WHO African region, and 14 (20%) were in the WHO South-East Asia Region, while 6 were located in the America, Eastern Mediterranean, or globally. 14/50 studies included the definition used for severe malnutrition: 6/14 defined a patient as severely malnourished when the weight for height falls below three standard deviations from the median of the WHO reference values, 6/14 as weight for height less than 70% of the median of WHO reference values, 1/14 reported both definitions, 1/14 defined patient malnourished when the weight for age < 60% and bilateral oedema was present. Thirty-four (49%) studies excluding severe malnutrition were in children < 15 years, 7/70 studies reported included patients aged above 15 years old, 24/70 did not include an upper age limit for enrolment, 5/70 did not define age limits for study inclusion. Other concomitant reasons for exclusion were: febrile illness other than malaria (55, 31%), heart impairment (30, 17%), current medication likely to interfere with therapy (21, 12%), history of convulsions (20, 11%), HIV infection (16, 9%), ECG abnormalities (9, 5%), previous history of splenectomy (9, 5%), recent blood transfusion (5, 3%), hepatitis (4, 2%), and sickle cell disease (4, 2%). One study specifically included patients who were only HIV positive. Further details on concomitant criteria are reported in Additional file 4.

### Clinical reasons

Patients experiencing severe vomiting or diarrhoea (39, 22%), impaired consciousness (12, 7%), and inability to stand up or eat (9, 5%) were excluded in 39/176, 12/176 and 9/176 trials, respectively. Seven out of 176 studies mentioned G6PD minimum level as criteria for inclusion, three of which were on P. *falciparum* and four on P. *vivax*. Three (2%) studies excluded patients with G6PD minimum level, one (0.6%) on P. *falciparum* and two (1%) on P. *vivax*. Other unspecified clinical reasons, respiratory distress, and haemoglobinuria were also reported in 5/176, 2/176, and 1/176 trial(s), respectively.

#### Laboratory values and cut-off levels—Haematology

Minimum cut-off levels were reported for haemoglobin, packed cell volume (PCV), or haematocrit in 65 (37%), five (3%), and five (3%) of the studies, respectively. The haemoglobin cut-off level was used as a criterion for 51/134 (38%) of *P. falciparum*, and 7/23 (30%) *P. vivax* trials. The cut-off levels were: for haemoglobin < 5 g/dL in 23/55 (42%) trials, < 6 g/dL in 3/55 (6%), < 7 g/dL in 15/55 (27%), < 8 g/dL in 12/55 (22%) studies and < 10 g/dL and < 11 g/dL in one publication (2%) each. Ten studies did not report a cut-off for exclusion. For PCV < 20% in 4 studies, and < 25% in one study; for haematocrit < 25% in four and < 18% in one trial. Low platelets patients were excluded in one (0.6%) study, no cut-off for exclusion was defined in the publication.

#### Laboratory values and cut-off levels—Biochemistry

Thirty-six (20%) trials excluded subjects with hepatic impairment: 29 for *P. falciparum*, five for *P. vivax*, one for mixed infection, and one for *P. knowlesi*. 3/36 publications included a definition for hepatic impairment: either as alanine aminotransferase (ALT) concentration 2.5-fold above the upper limit of normal (1/3 studies) or more than twice the upper limit of normal (2/3). Thirty-six (20%) trials excluded subjects with renal impairment: 31 on *P. falciparum*, three on *P. vivax,* one on mixed infection, and one on *P. knowlesi.* Only one study defined renal impairment as creatinine level above 1.5 times the upper limit of normal. Abnormal creatine phosphokinase, glucose level and electrolyte imbalance were exclusion criteria in one (0.6%) study per criteria.

#### Operational reasons

Eleven percent (19/176) of the trials excluded patients participating in another research study. 14 (8%) studies excluded patients for operational reasons without reporting a more specific motive. Patients that were lost during the screening process, who had recently travelled beyond their hometown, or withdrawn were excluded in four (2%), one (0.6%), and one (0.6%) of the trials, respectively. Problems with mosquito husbandry (one, 0.6%) were also reported as exclusion criteria.

#### Other criteria

Non-descriptive criteria like ‘not specified’ were reported as the criteria in patients in 42 (24%) studies.

### Reported reasons for exclusions of malaria-positive subjects

The 176 studies, which collectively screened 553,300 suspected malaria cases, identified as malaria-positive 157,516 (28%), of whom 56,293 (36%) were enrolled into the trial and treated, while 101,223 (64%) were excluded.

The reason for excluding those who tested positive for malaria was reported for 72,226/101,223 (71%). Three criteria account for 69.5% of exclusions: mixed-species malaria infections or other specific *Plasmodium* species (35.5%); parasite counts outside the set study ranges (20.5%); and refusal of consent (13.5%) (Table 3). The remaining studies did not specify reasons for exclusion, corresponding to 28,921 (29%) unaccounted exclusions.

**Table 2:** Top 10 causes of exclusion malaria-positive patients. Other reasons and corresponding number of excluded patients are reported in Additional file 5

Reasons for exclusion	No. of subjects excluded- reasons described	% exclude—reasons described
Presence of mixed infections or specific plasmodium species	25,661	35.51%
Parasitaemia	14,780	20.45%
Refusal of consent	9790	13.55%
Not Specified^a^	5952	8.23%
Agreement to follow-up	2941	4.07%
Operational reasons^b^	2664	3.69%
Age	1319	1.83%
History of fever or current fever	1230	1.70%
Recent anti-malarial drug use and anti-malarial vaccine	1103	1.53%
Proximity to accommodation	1096	1.52%

^a^Such patients have been explicitly excluded from the studies of interest without being specifically reported the reason for exclusion

^b^“Operational reasons” is a generic terminology reported in several papers

### Comparison of the baseline characteristics and planned eligibility criteria

Baseline demographics of the 176 studies were extracted from the study baseline demographics table, or the text when available. When presented in the paper, the baseline demographics were then compared with the study eligibility criteria range (Table 4).

**Table 3:** Comparison of the actual baseline characteristics with the criteria for eligibility

Criteria	No. of studies Reporting the criteria ranges/cut-off	No. of studies reporting criteria ranges/cut-off and baseline characteristics (%)^a^	No. of studies reporting participant(s) outside the accepted inclusion ranges/cut-off (%)^b^
Age	151	60 (40)	5 (8)
Parasitaemia	136	61 (45)	21 (34)
Haemoglobin level	55	10 (18)	4 (40)
Temperature	118	29 (25)	23 (80)
Weight	52	16 (31)	0 (0)
Haematocrit	4	0 (0)	–
Packed Cell Volume	5	1 (20)	1 (20)
Hepatic impairment (ALT)	3	0 (0)	–
Renal impairment (Creatinine)	1	0 (0)	–

^a^The percentages represent the proportion of the studies reporting criteria ranges/cut-off and baseline characteristics over the number of studies reporting the criteria

^b^The percentages represent the proportion of the studies enrolling at least a patient outside the accepted inclusion range/cut-off definition over the studies reporting criteria ranges/cut-off and baseline characteristics

#### Age

Of the 151 studies reporting age as an inclusion criterion, 60 (40%) reported both the eligible age and the actual baseline age range. Of those studies that reported both eligible and baseline age, 26 (43%) reported only a minimum age limit for inclusion in the study (no upper limit). One of those 60 studies enrolled one or more patient(s) aged below the defined cut-off. Four of the 60 studies included participants aged above the pre-defined ranges.

#### Parasitaemia

For this analysis, we separated the parasite counts by species as *P*. *falciparum* and *P*. *vivax*. Forty-five percent of studies (61/136) that reported parasitaemia inclusion ranges also reported actual baseline ranges: 52 (85%) of these were for *P. falciparum* (and mixed species including *P. falciparum*), 10 were for *P*. *vivax* and one for *P. knowlesi.* Of the 52 *P. falciparum* studies, 18 did not have an upper limit for inclusion. Of the 34 studies with an eligible parasite count range, 14 (41%) had a baseline parasitaemia range that fell outside the study eligible range, with eight of them enrolling patients with counts exceeding the maximum value allowed for inclusion in the study. Seven of the *P. vivax* studies which reported baseline parasitaemia ranges did not report an upper limit for study parasite count inclusion criteria. One of the *P*. *vivax* studies had a baseline parasitaemia range that fell outside the study inclusion criteria ranges.

#### Haemoglobin level

The range of haemoglobin cut-off minimum level for exclusion reported in the studies was from  ≤ 11 g/dL to < 5 g/dL. In 10/176 (5%) of studies both a haemoglobin minimum cut-off level for exclusion and baseline haemoglobin range were reported, of which eight studies were for *P*. *falciparum* and two were on *P. vivax*. Four of those 10 studies enrolled one or more patient(s) with a haemoglobin level below the defined cut-off.

Other baseline characteristics collected are reported in Table 4.

## Discussion

This is the first review to address the question of the representativeness of the patients enrolled in malaria treatment trials with respect to the spectrum of patients seen in clinical practice. This effort identified 176 studies conducted over the last two decades, screening over 150,000 patients from which both the eligibility criteria adopted and how these translated into patient selection could be extracted. About two-thirds of the malaria-positive subjects screened for enrolment are systematically excluded from anti-malarial treatment trials, and the reason for almost one-third of them was not reported.

This analysis points also to an even larger body of information which is currently not available, largely because basic reporting rules are not adhered to, leading to a substantial erosion of the mass of information potentially available. These 176 studies represent about one-fifth of the articles on the treatment of uncomplicated malaria which could have contributed to this analysis; however, it is unclear whether the desired information in the remaining approximately four-fifths of studies was not collected, not recorded, or simply not reported by the authors. Although this analysis was restricted to studies published after the CONSORT 2001 [12] statement, only just over half of the malaria clinical trials conducted between 2001 and 2021 (556/847) present the CONSORT flow diagram or an adaptation thereof, or produce similar information.

The study population from these trials consists of parasitological and non-parasitological confirmed uncomplicated malaria, either symptomatic or asymptomatic, across *Plasmodium* species of either mono- or mixed-infection. Severe malaria patients are generally excluded, either explicitly or by excluding parasite count levels defining more complicated malaria cases. This is also due to the eligibility criteria defined in the review which did not include studies investigating severe malaria patients. Studies also covered a broad age range, with more than one-fifth of the enrolled subjects being under five years of age.

The WHO guidelines [3], which aim to standardize the assessment of treatment efficacy primarily to monitor resistance in the most reproducible and consistent manner possible, require stringent eligibility criteria when measuring an outcome of interest, especially drug efficacy in terms of parasite and fever clearance in subjects with no or little immunity to help clear the parasites [21]. The current analysis identified 84 main criteria (17 inclusions, 75 exclusions, 8 reported in both sections) from the 176 studies assessed which generally complied with the guidelines, though to various degrees.

In the studies reporting the number of subjects excluded due to a specific reason, three criteria account for about 70% of exclusion of malaria-positive patients in studies, although some patients could have been excluded for not meeting more than one criterion. One set of reasons (more than half of exclusions) relates to parasitological diagnosis requiring microscopy—mixed or other infections and parasitaemia outside the established range.

As currently designed, anti-malarial efficacy trials do not provide information on how patients with higher parasitaemia and no obvious sign of severe malaria would respond to oral treatment, as well as participants with other concomitant diseases or other clinical reasons. Conversely, trials seem to cover adequately subjects with all degrees of anaemia except severe anaemia defined as haemoglobin < 5 g/dl. More detailed information on patients with high parasitaemia and/or severely anaemic would be desirable.

Some inconsistencies also emerged between the study criteria ranges or cut-off levels for eligibility and the related baseline characteristics of the recruited patients. Among the criteria analysed, temperature, parasitaemia and age ranges are poorly reported across the studies, although this inconsistency could be due to the timing at which the value is measured, with a variation in the value according to whether the measurement occurred at the screening or the baseline.

## Limitations

The criteria were extracted and grouped according to how they were reported in the publications. As also demonstrated by the poor definition of some criteria (e.g., hepatic and renal impairment) or the difference in definitions detected (e.g., severe malnutrition), there is no standardized definition for each criteria reported, which will likely make the profile of the study populations heterogenous. The search strategy of this review did not include the grey literature and 12% of the peer-reviewed articles were excluded as they could not be accessed. Nonetheless, the sizable number of studies identified and number of participants give confidence in the evidence of knowledge gaps on the generalizability of malaria clinical trials. It is possible that some studies were missed, also considering that the update search was performed using only the WWARN Publication library [11], as the iterative approach used might have created some discrepancy in the search methods used. While it was not possible to report separately on phase II, III or IV studies as this information was not provided systematically, it is reasonable to assume that they were mostly “phase IV-type” studies as they tested therapies in routine use; phase II or III aimed at supporting drug registrations were clearly identified and limited in numbers.

Accurate reporting is essential. The CONSORT [10] statement offers a useful framework for improving the transparency of clinical trials, including adequate reporting of the eligibility criteria and patient attrition. Papers should report details on the reasons for excluding subjects against the pre-established eligibility criteria; trial screening logs could be an important source of information. Recording and sharing this information would allow a better profiling of the patients seen in routine practice and a better understanding of the representativeness of the body of evidence from clinical trials. A number of tools have been made available to the research community and should be further disseminated to improve the quality of anti-malarial efficacy trials and their reporting [22, 23].

## Conclusion

Research findings are summarized in Box 5. The studies identified generally adhere to the WHO standards, which are important for assessing drug efficacy in a consistent way [3]. At the same time, pragmatic (effectiveness) trials are clearly lacking, and these too are important, as they would provide critical, generalizable information on patient groups who are systematically excluded from efficacy trials but are routinely seen and treated. The purpose of such evaluation is different, and the selection criteria established for monitoring resistance applied may limit the external validity of results for the general population and the conditions in which treatments are delivered in practice.

**Box 2:** Research in context and added value of the study

Evidence before this study: This is the first review to address the question of the representativeness of the patients enrolled in malaria treatment trials with respect to the population of patients seen in clinical practice, covering the last two decades. No relevant previous study conducted on this subject could be identified after searching MEDLINE and the Cochrane Library
Added-value of this study: The findings of this study provide evidence of knowledge gaps on the generalizability of current malaria clinical efficacy trials to the general population. The study identifies and quantifies systematic exclusions based on parasite counts (especially high parasitaemia) and parasite species (in particular mixed infections); these patients will be treated in practice but are under-researched in clinical trials. There was also significant under-reporting of the reason for exclusions of malaria-positive patients in trials
Implications of all the available evidence: These results point to three main needs: conducting pragmatic (effectiveness) trials to capture the full range of cases treated in real-life; filling the knowledge gap on the treatment of mixed infections; and providing accurate reporting of exclusions. The CONSORT statement offers a valuable framework for improving the transparency of clinical trials, including adequate reporting of the eligibility criteria and of patient attrition. Papers should report details on the reasons for excluding subjects against pre-established eligibility criteria, for which trial screening logs could be an important source of information. Recording and sharing this information would allow better profiling of the patients seen in routine practice and a better understanding of the representativeness of the body of evidence from clinical trials

## Supplementary Information

**Additional file 1. Dataset. Spreadsheet of malaria trials eligibility criteria.**

**Additional file 2. Variable Dictionary. **

**Additional file 3. PubMed search terms. **

**Additional file 4. Criteria group summary. **

**Additional file 5.** Reasons for exclusion summary analysis.

**Additional file 6.** Details of 176 articles included in the final assessment.

**Additional file 7.** Data items, baseline demographics, and subgroup narrative analysis details.

**Additional file 8.** Yearly and cumulative number of studies by Plasmodium species between 2001-2021 for 176 studies.

**Additional file 9.** Age ranges distributions across the studies reporting Age as inclusion criteria.

**Additional file 10.** Temperature (°C) minimum requirement for enrolment across the studies reporting Temperature as inclusion criteria.

## Data Availability

All data generated or analysed during this study are included in this published article and its Additional files.

## Abbreviations

WWARN: WorldWide antimalarial resistance network
WHO: World Health Organization
PRISMA: Preferred reporting items for systematic reviews and meta-analyses extension
MESH: Medical subject headings
CONSORT: Consolidated standards of reporting trials
RCT: Randomized controlled trial
PCR: Polymerase chain reaction
RDT: Rapid diagnostic test
G6PD: Glucose-6-Phosphate dehydrogenase
PCV: Packed cell volume

## References

[1] WHO (2021). World malaria report 2021.

[2] WHO (2021). Guidelines for malaria.

[3] Van Spall HG, Toren A, Kiss A, Fowler RA (2007). Eligibility criteria of randomized controlled trials published in high-impact general medical journals: a systematic sampling review. JAMA.

[4] Dekkers OM, von Elm E, Algra A, Romijn JA, Vandenbroucke JP (2010). How to assess the external validity of therapeutic trials: a conceptual approach. Int J Epidemiol.

[5] Nsanzabana C, Djalle D, Guérin PJ, Ménard D, González IJ (2018). Tools for surveillance of anti-malarial drug resistance: an assessment of the current landscape. Malar J.

[6] WHO (2009). Method for surveillance of antimalarial drug efficacy.

[7] Bassat Q, González R, Machevo S, Nahum A, Lyimo J, Maiga H (2011). Similar efficacy and safety of artemether-lumefantrine (Coartem^®^) in African infants and children with uncomplicated falciparum malaria across different body weight ranges. Malar J.

[8] Moher D, Liberati A, Tetzlaff J, Altman DG, PRISMA Group (2009). Preferred reporting items for systematic reviews and meta-analyses: the PRISMA statement. BMJ.

[9] Worldwide Antimalarial Resistance Network. WWARN Clinical Trials Publication Library. 2021. https://www.wwarn.org/tools-resources/literature-reviews/wwarn-clinical-trials-publication-library. Accessed 28 Mar 2021

[10] Schulz KF, Altman DG, Moher D, CONSORT Group (2010). CONSORT 2010 Statement: Updated guidelines for reporting parallel group randomized trials. Ann Intern Med.

[11] Takata J, Sondo P, Humphreys GS, Burrow R, Maguire B, Hossain MS (2020). The worldwide antimalarial resistance network clinical trials publication library: a live, open-access database of plasmodium treatment efficacy trials. Am J Trop Med Hyg.

[12] Moher D, Schulz KF, Altman DG (2001). The CONSORT statement: revised recommendations for improving the quality of reports of parallel group randomized trials. BMC Med Res Methodol.

[13] Harris PA, Taylor R, Thielke R, Payne J, Gonzalez N, Conde JG (2010). Research electronic data capture (REDCap)—a metadata-driven methodology and workflow process for providing translational research informatics support. J Biomed Inform.

[14] Harris PA, Taylor R, Minor BL, Elliott V, Fernandez M, Neal LO (2019). The REDCap consortium: building an international community of software platform partners. J Biomed Inform.

[15] WHO (1996). Assessment of therapeutic efficacy.

[16] WHO (2002). Monitoring antimalarial drug resistance.

[17] WHO (2003). Assessment and monitoring of antimalarial drug efficacy for the treatment of uncomplicated Falciparum Malaria.

[18] Sutherland CJ, Drakeley CJ, Obisike U, Coleman R, Jawara M, Targett GAT (2003). The addition of artesunate to chloroquine for treatment of *Plasmodium falciparum* malaria in Gambian children delays, but does not prevent treatment failure. Am J Trop Med Hyg.

[19] Valecha N, Phyo AP, Mayxay M, Newton PN, Krudsood S, Keomany S (2010). An open-label, randomised study of dihydroartemisinin-piperaquine versus artesunate-mefloquine for falciparum malaria in Asia. PLoS ONE.

[20] Secretaria de Salud de Honduras. Norma de Malaria en Honduras. Secretaria de Salud SdRP, Direccion General de Promocion de la Salud, Programa Nacional de la Prevencion y Control de la Malaria. Tegucigalpa, Honduras: Secretaria de Salud de Honduras, 2010.

[21] Allen EN, Chandler CIR, Mandimika N, Pace C, Mehta U, Barnes KI (2013). Evaluating harm associated with anti-malarial drugs: a survey of methods used by clinical researchers to elicit, assess and record participant-reported adverse events and related data. Malar J.

[22] Worldwide Antimalarial Resistance Network. Malaria Clinical Trials Toolkit. https://www.wwarn.org/tools-resources/malaria-clinical-trials-toolkit. Accessed 28 Mar 2022

[23] Global Health Trial Network. Global Drug Trials. https://globaldrugdevelopment.tghn.org/ Accessed 28 Mar 2022

