# Supplementary material for: Malaria patient spectrum representation in therapeutic clinical trials of uncomplicated malaria: a scoping review of the literature
Source: Malar J. 2023 Feb 10;22:50. doi: 10.1186/s12936-023-04441-5 (PMC9913008; doi:10.1186/s12936-023-04441-5)
Supplement: Supplementary file 3 — Additional file 3. PubMed search terms. [file 12936_2023_4441_MOESM3_ESM.docx]

Therapy/Narrow[filter] AND (("malaria"[MeSH Terms] OR "malaria"[All Fields]) AND ("therapy"[Subheading] OR "therapy"[All Fields] OR "treatment"[All Fields] OR "therapeutics"[MeSH Terms] OR "therapeutics"[All Fields])) AND (Clinical Trial[ptyp] AND ("2001/04/14"[PDAT] : "2017/12/31"[PDAT]))

Therapy/Narrow[filter] AND ("antimalarials"[Pharmacological Action] OR "antimalarials"[MeSH Terms] OR "antimalarials"[All Fields] OR ("antimalarial"[All Fields] AND "drugs"[All Fields]) OR "antimalarial drugs"[All Fields]) AND ("2001/04/14"[PDAT] :

Therapy/Narrow[filter] AND ("antimalarials"[Pharmacological Action] OR "antimalarials"[MeSH Terms] OR "antimalarials"[All Fields] OR ("antimalarial"[All Fields] AND "drugs"[All Fields]) OR "antimalarial drugs"[All Fields]) AND ("2001/04/14"[PDAT] : "2017/12/31"[PDAT])

**Terms-**Malaria treatment; Antimalarials/therapeutic use*; Malaria/drug therapy*;Antimalarials/administration & dosage*;Drug Therapy; Combination;
