# Supplementary material for: Malaria patient spectrum representation in therapeutic clinical trials of uncomplicated malaria: a scoping review of the literature
Source: Malar J. 2023 Feb 10;22:50. doi: 10.1186/s12936-023-04441-5 (PMC9913008; doi:10.1186/s12936-023-04441-5)
Supplement: Supplementary file 7 — Additional file 7. Data items, baseline demographics, and subgroup narrative analysis details. [file 12936_2023_4441_MOESM7_ESM.docx]

*Data items*: information on the author, title, language and study design characteristics such as publication date, location, and the WHO geographical region was extracted. Where multiple sites were reported in the study the WHO Region was listed as “Global”. Antimalarial medication type, randomization, blinding, and follow-up period were also reported. We extracted the Plasmodium species examined in the trial. A study that included more than one species was classified as a mixed publication in the posterior analysis. Information was also collected on whether a reference was provided for the eligibility criteria, as well as trial population data such as the total number of patients screened, the total number included and excluded, the total number of patients malaria-positive screened and the total number malaria-positive excluded. We extracted the eligibility criteria, including inclusion criteria and exclusion criteria, and the number of excluded patients per each criterion, when reported. For cross-over trials eligible for inclusion in the study, only data from the initial segment of the trial was used, to limit the carry-over effects.

*Baseline demographics:* baseline demographics were also recorded, including the mean (when available), median, and range, for use as a proxy for the general population demographics, and as a comparator to the subjects eligible for the clinical trial. Key terms from this dictionary are included in Supplementary file 1.

*Data validation*: the dataset was populated using information extracted from the text and CONSORT Flow Diagram. The dataset was then checked systematically for all the variables. Subsequently, the eligibility criteria were gathered in different groups. The group selection and definition are reported in Supplementary file 2.

*Subgroup narrative analysis: a* systematic narrative synthesis was performed on the eligibility criteria. The narrative analysis also explored the concordance between these exclusion criteria and baseline demographics.
