# Supplementary material for: Malaria patient spectrum representation in therapeutic clinical trials of uncomplicated malaria: a scoping review of the literature
Source: Malar J. 2023 Feb 10;22:50. doi: 10.1186/s12936-023-04441-5 (PMC9913008; doi:10.1186/s12936-023-04441-5)
Supplement: Supplementary file 8 — Additional file 8. Yearly and cumulative number of studies by Plasmodium species between 2001-2021 for 176 studies. [file 12936_2023_4441_MOESM8_ESM.docx]

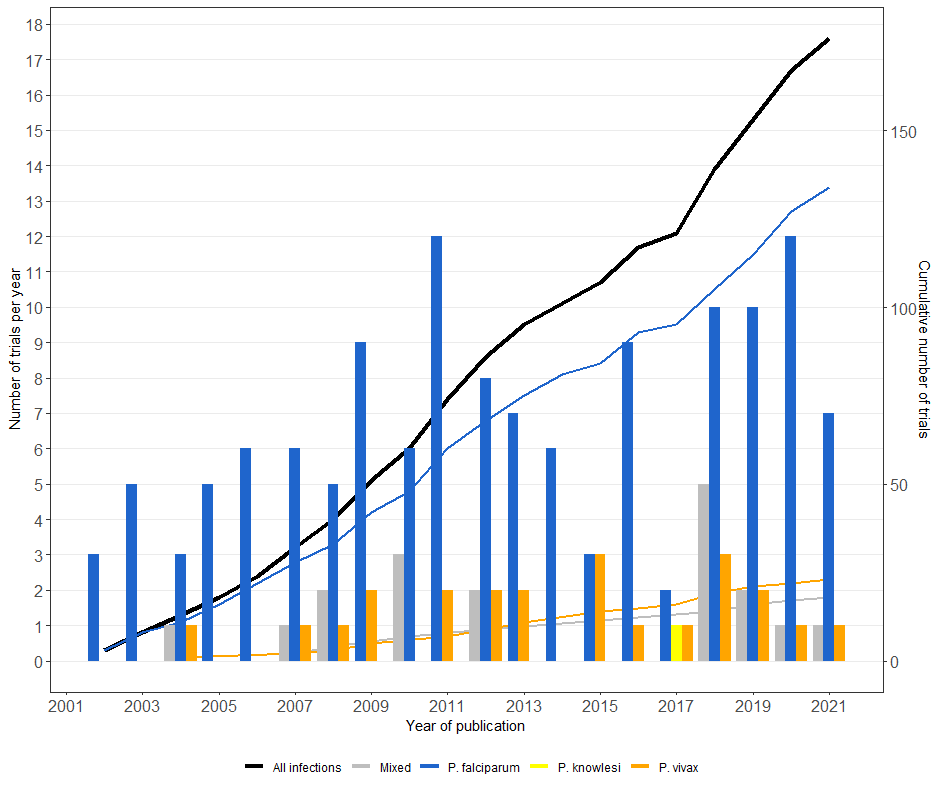


*Figure Additional file 8. Yearly and cumulative number of studies by Plasmodium species between 2001-2021 for 176 studies. The bars represent the number of publications published each year by species (left Y axis); the lines represent the cumulative number of publications by species studied and the total cumulative number of studies (right Y axis)*
