# Supplementary material for: Malaria patient spectrum representation in therapeutic clinical trials of uncomplicated malaria: a scoping review of the literature
Source: Malar J. 2023 Feb 10;22:50. doi: 10.1186/s12936-023-04441-5 (PMC9913008; doi:10.1186/s12936-023-04441-5)
Supplement: Supplementary file 9 — Additional file 9. Age ranges distributions across the studies reporting Age as inclusion criteria. [file 12936_2023_4441_MOESM9_ESM.docx]

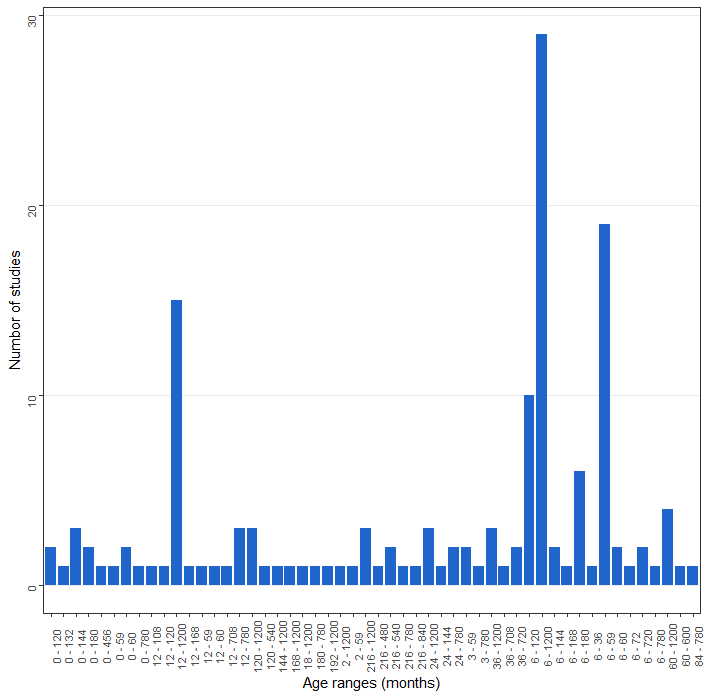


*Figure Additional file 9. Age ranges distributions across the studies reporting Age as inclusion criteria.*
