# Supplementary material for: Malaria patient spectrum representation in therapeutic clinical trials of uncomplicated malaria: a scoping review of the literature
Source: Malar J. 2023 Feb 10;22:50. doi: 10.1186/s12936-023-04441-5 (PMC9913008; doi:10.1186/s12936-023-04441-5)
Supplement: Supplementary file 10 — Additional file 10. Temperature (°C) minimum requirement for enrolment across the studies reporting Temperature as inclusion criteria. [file 12936_2023_4441_MOESM10_ESM.docx]

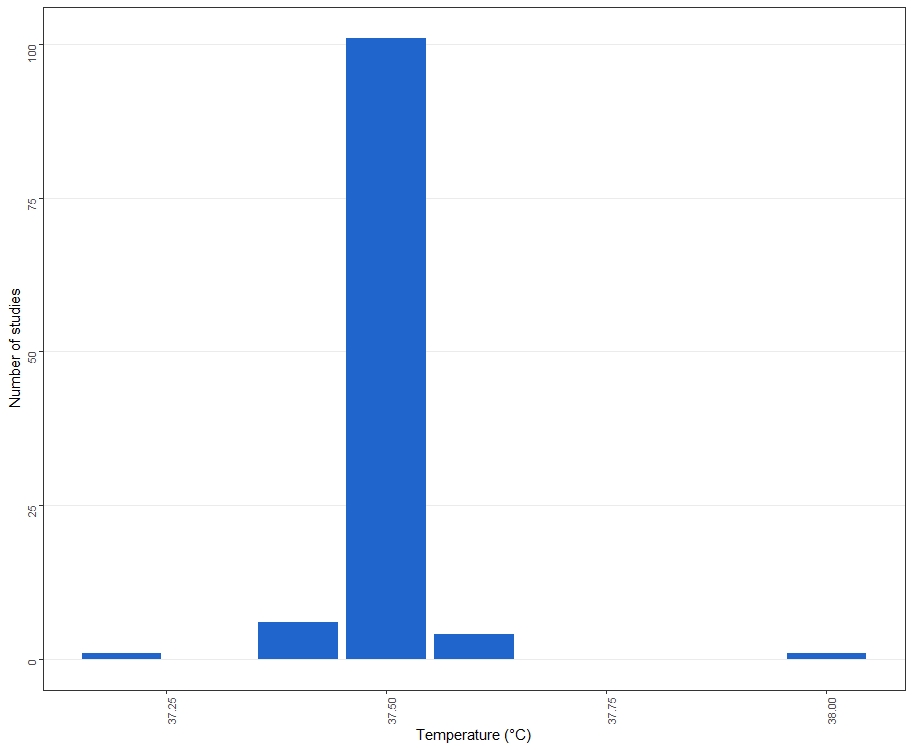


*Figure Additional file 10. Temperature (°C) minimum requirement for enrolment across the studies reporting Temperature as inclusion criteria.*
